# FreeSurfer and 3D Slicer-Assisted SEEG Implantation for Drug-Resistant Epilepsy

**DOI:** 10.3389/fnbot.2022.848746

**Published:** 2022-02-28

**Authors:** Qiangqiang Liu, Junjie Wang, Changquan Wang, Fang Wei, Chencheng Zhang, Hongjiang Wei, Xiaolai Ye, Jiwen Xu

**Affiliations:** ^1^Department of Neurosurgery, Clinical Neuroscience Center Comprehensive Epilepsy Unit, Ruijin Hospital, Shanghai Jiao Tong University School of Medicine, Shanghai, China; ^2^Clinical Neuroscience Center, Ruijin Hospital Luwan Branch, Shanghai Jiao Tong University School of Medicine, Shanghai, China; ^3^Wuhan Zhongke Industrial Research Institute of Medical Science Co., Ltd., Wuhan, China; ^4^Department of Neurosurgery, Center for Functional Neurosurgery, Ruijin Hospital, Shanghai Jiao Tong University School of Medicine, Shanghai, China; ^5^Shanghai Research Center for Brain Science and Brain-Inspired Intelligence, Shanghai, China; ^6^School of Biomedical Engineering, Shanghai Jiao Tong University, Shanghai, China; ^7^Institute of Medical Robotics, Shanghai Jiao Tong University, Shanghai, China

**Keywords:** stereoelectroencephalography (SEEG), robotics, stereotactic neurosurgery, FreeSurfer, 3D Slicer

## Abstract

**Objective:**

Our study aimed to develop an approach to improve the speed and resolution of cerebral-hemisphere and lesion modeling and evaluate the advantages and disadvantages of robot-assisted surgical planning software.

**Methods:**

We applied both conventional robot planning software (method 1) and open-source auxiliary software (FreeSurfer and 3D Slicer; method 2) to model the brain and lesions in 19 patients with drug-resistant epilepsy. The patients' mean age at implantation was 21.4 years (range, 6–52 years). Each patient received an average of 12 electrodes (range, 9–16) between May and November 2021. The electrode-implantation plan was designed based on the models established using the two methods. We statistically analyzed and compared the duration of designing the models and planning the implantation using these two methods and performed the surgeries with the implantation plan designed using the auxiliary software.

**Results:**

A significantly longer time was needed to reconstruct a cerebral-hemisphere model using method 1 (mean, 206 s) than using method 2 (mean, 20 s) (*p* < 0.05). Both methods identified a mean of 1.4 lesions (range, 1–5) in each patient. Overall, using method 1 required longer (mean, 130 s; range, 48–436) than using method 2 (mean, 68.1 s; range, 50–104; *p* < 0.05). In addition, the clarity of the model based on method 1 was lower than that based on method 2. To devise an electrode-implantation plan, it took 9.1–25.5 min (mean, 16) and 6.6–14.8 min (mean, 10.2) based on methods 1 and 2, respectively (*p* < 0.05). The average target point error of 231 electrodes amounted to 1.90 mm ± 0.37 mm (range, 0.33–3.61 mm). The average entry point error was 0.89 ± 0.26 mm (range, 0.17–1.67 mm). None of the patients presented with intracranial hemorrhage or infection, and no other serious complications were observed.

**Conclusions:**

FreeSurfer and 3D Slicer-assisted SEEG implantation is an excellent approach to enhance modeling speed and resolution, shorten the electrode-implantation planning time, and boost the efficiency of clinical work. These well-known, trusted open-source programs do not have explicitly restricted licenses. These tools, therefore, seem well suited for clinical-research applications under the premise of approval by an ethics committee, informed consent of the patient, and clinical judgment of the surgeon.

## Introduction

Stereoelectroencephalography (SEEG) utilizes deep electrodes implanted under stereotactic guidance to explore deep cortical and intra-sulcal structures and construct a three-dimensional (3D) map of the epileptogenic zone (Isnard et al., 2018). The effectiveness of SEEG depends on the quality of the electrode-implantation plan. Robot-assisted SEEG electrode implantation is increasingly being performed worldwide; numerous reports on its safety and accuracy have been published in the literature (Abel et al., 2018; Spyrantis et al., 2019; Bonda et al., 2021).

Establishing cerebral-hemisphere and lesion models conveniently and accurately is very important for electrode implantation. FreeSurfer and 3D Slicer are well-established open-source programs that can be freely used. FreeSurfer can reconstruct left and right pail models of brain, and even segmentation of special regions of brain (such as primary motor cortex, Broca area) (Dale et al., 1999; Fischl, 2012). 3D Slicer have a number of editor effects for model reconstruction, and segmentation may be performed manually, semi-automatic or even fully automatic (Fedorov et al., 2012; Kapur et al., 2016).

Some studies have applied Freesurfer, 3D Slicer and automated multiple trajectory planning algorithm to compute trajectory of electrode (De Momi et al., 2013; Sparks et al., 2017; Vakharia et al., 2019). These methods could automatically avoid blood vessels or important structures of brain, increase the coverage of gray matter and compute the entry angle of electrode in the skull. These methods focused on the safety of SEEG implantation, but the lesion models were ignored and didn't help with epileptologists to define a preliminary electrode-implantation plan. Recently, a study proposed a multimodal and multidisciplinary platform for electrode-implantation plan and postoperative analysis (Higueras-Esteban et al., 2021). In this study, a variety of methods including FreeSurfer segmentation were used to evaluate the SEEG plans of 19 patients, and were helpful for the defining and validating of SEEG plans. But the lesion models were also ignored, and the transfer step of SEEG plans from platform to robotic system were not conveniently. In addition, several studies have applied 3D Slicer to the analysis and visualization of SEEG contacts (Princich et al., 2013; Narizzano et al., 2017).

To explore the feasibility and efficiency of the use of these tools in clinical practice, this study evaluated the outcomes of robot-assisted FreeSurfer and 3D Slicer-assisted SEEG implantation in 19 consecutive patients with drug-resistant epilepsy.

## Methods

### Study Participants

Our study included a cohort of 19 consecutively enrolled patients who underwent SEEG implantation at the epilepsy centers of the Ruijin Hospital in China between May and November 2021. All cases were discussed at a multidisciplinary epilepsy conference, and all patients were diagnosed with drug-resistant epilepsy and underwent intracranial monitoring thereafter.

### Neuroimaging Acquisition

All patients underwent standardized positron emission tomography (PET) magnetic resonance (MR) scanning, which consisted of T1 magnetization-prepared rapid acquisition gradient echo (MPRAGE), T2-weighted fluid-attenuated inversion recovery (Flair), and fluorodeoxyglucose PET imaging. The patients selected for SEEG electrode implantation underwent head computed tomography (CT) angiography.

### Surgical Planning

The surgical planning consisted of two steps: model establishment and electrode-implantation plan. The models were reconstructed using two different methods: (1) the surgical planning software of the robotic system, and (2) FreeSurfer (version 7.1) and 3D Slicer (version 4.10.2) software to model the bilateral cerebral hemispheres and lesions, respectively (Fischl, 2012; Kapur et al., 2016). The established models were then used to construct an electrode-implantation plan. For each method, the time required to establish the model and design the corresponding electrode-implantation plan was analyzed. Modeling and electrode-implantation planning were performed by the same physician.

#### Modeling Method 1

The Sinovation surgical planning system (Sinovation, Beijing, China) was used to model the brains and lesions of our patients. All imaging data were imported into planning system. A unilateral cerebral hemisphere model was created for patients who required unilateral electrode implantation, whereas a bilateral cerebral hemisphere model was created for patients who required bilateral electrode implantation. At present, surgical planning systems include limited modeling tools but provide basic functions, such as thresholding, volume rendering, and clipping. We used volume rendering to establish a whole-brain model base on the Flair images and then manually cropped off the scalp, blood vessels, and contralateral hemisphere with the cropping tool. The lesion models were also cut from whole-brain model.

#### Modeling Method 2

For method 2, we utilized open-source programs for modeling. The “recon-all” command of FreeSurfer was utilized to reconstruct a bilateral brain surface model from the T1 MPRAGE images. This calculation process required 4–7 h but was fully automatic. In our preoperative evaluation process, this step will be completed before the preoperative discussion. Then, pial surfaces (lh.pail and rh.pail) form FreeSurfer and imaging data were simultaneously imported into 3D Slicer and combined with structural magnetic resonance and PET images. The modular of Segment Editor was used to model suspected epileptic lesions according to signal and morphology of images. After all models were reconstructed, they were converted into Digital Imaging and Communications in Medicine (DICOM) format using the modular of DICOM Export. Finally, because of the 3D Slicer and Sinovation surgical planning system used the same reference images (T1 MPRAGE images), the cerebral-hemisphere and lesion models could import into the surgical planning system directly without additional registration. The conversion step of cerebral hemisphere model required 20 s, so we accordingly set the cerebral hemisphere modeling time to 20 s. The time required to establish the lesion model was calculated separately.

#### Electrode-Implantation Plan

The Sinovation surgical planning system was utilized to create an electrode-implantation plan according to the results of the preoperative discussion. The electrode-implantation draft was designed in template space (similar to Talairach grid) by epileptologist. In the planning system, the draft was transformed into an actual implantation scheme by one neurosurgeon. Memory effect did exist to some extent, but this work depended on the precise understanding of the models, images and anatomy. These knowledges were different among different doctors, so we didn't use several doctors. In order to reduce the bias, the plan was designed based on the method 2 firstly, and then method 1. The designing time was calculated separately. The same vascular models were used, so the time to avoid blood vessels was not calculated.

### Robot-Guided SEEG Electrode Implantation

**Figure 1:**
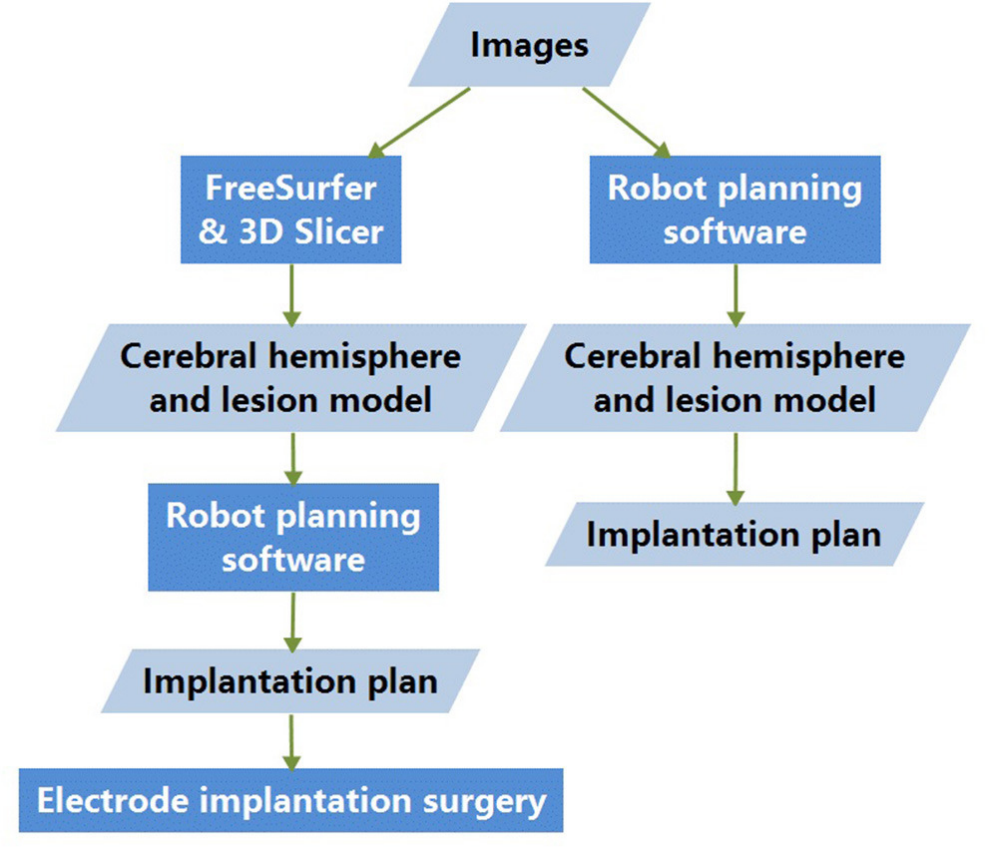
Basic research process. Images are processed with both methods 1 and 2; the difference lies in the cerebral-hemisphere and lesion-model generation. The electrode-implantation surgeries were conducted based on the model and implantation plan designed with method 2.

All the procedures were performed using the same Sinovation robotic system. Electrodes (HKHS, Beijing, China) were implanted in a standardized manner, as previously described (Zhao et al., 2020). All patients underwent intraoperative CT scanning to verify the position of each electrode and to identify any signs of intracranial hemorrhage immediately after implantation. The accuracy of each stereotactic electrode was confirmed by fusing pre-implantation MR and post-implantation CT images. The initially planned position and the actual position of the electrodes were compared, and the target point error (TPE) was calculated. The accuracy was determined by calculating the TPE along with the entry point error (EPE) by applying the Euclidean distance, as described in previous studies (Kelman et al., 2010; Spyrantis et al., 2018). The basic research process is shown in Figure 1.

### Statistical Analysis

Differences in outcomes between the two methods were assessed using independent-samples *t*-tests for continuous variables and chi-square tests for categorical variables. Statistical significance was defined as a two-tailed *P*-value < 0.05. All statistical analyses were performed using Stata 11 (StataCorp, College Station, TX, USA).

## Results

### Patient Data

Nineteen patients underwent robot-assisted SEEG electrode implantation at Ruijin Hospital Luwan Branch. All procedures were performed by the same surgeon (Qiangqiang Liu). The patients' mean age at implantation was 21.4 years (range, 6–39 years). A total of 231 electrodes were implanted using the robot-assisted technique. Each patient received an average of 12 electrodes. Fourteen implantations were unilateral and included 7 left-cerebral and 7 right-cerebral implantations. Five implantations were bilateral, of which two were predominantly left sided. There were no postoperative complications, such as bleeding or infection. Tables 1, 2 present the demographic data and the results of the implantation procedure for each patient.

**Table 1:** Demographics and results of the implantation procedure for each study participant.

**Number**	**Gender**	**Age (years)**	**Laterality**	**Number of lesions**	**Number of electrodes**
1	Female	12	Unilateral (right)	1	15
2	Male	15	Bilateral	2	16
3	Female	9	Unilateral (right)	1	13
4	Female	39	Bilateral	1	11
5	Female	8	Unilateral (left)	1	12
6	Female	20	Unilateral (left)	1	11
7	Female	6	Unilateral (left)	1	9
8	Male	15	Unilateral (left)	5	12
9	Female	31	Bilateral	1	12
10	Female	32	Unilateral (right)	1	14
11	Male	19	Bilateral	2	12
12	Female	12	Bilateral	1	15
13	Male	24	Unilateral (left)	1	11
14	Female	52	Unilateral (left)	1	11
15	Female	19	Unilateral (left)	1	10
16	Female	7	Unilateral (right)	1	12
17	Male	9	Unilateral (left)	1	13
18	Female	48	Unilateral (right)	1	10
19	Female	30	Unilateral (right)	2	12
Mean		21.4		1.6	12.2

**Table 2:** Time to model and complete electrode implantation planning using methods 1 and 2.

**Number**	**Time to completion of**	**Time to completion**	**Time to completion of**	**Time to completion of**	**Electrode**	**Electrode**
	**of unilateral**	**of contralateral**	**of lesion**	**of lesion**	**implantation**	**implantation**
	**hemisphere**	**hemisphere**	**modeling**,	**modeling**,	**planning**,	**planning**,
	**modeling, method 1 (s)**	**modeling, method 1 (s)**	**method 1 (s)**	**method 2 (s)**	**method 1 (min)**	**method 2 (min)**
1	175.3	–	94.9	64.9	21.8	13.6
2	197.5	188.9	435.9	103.8	25.5	14.8
3	235.5	–	74.8	57.3	9.4	9.3
4	204.3	211.8	94.3	50.1	9.1	6.6
5	187.2	–	79.9	51.9	13.5	10.8
6	253.2	–	110.3	76.7	17	13.9
7	245.7	–	130.9	98.4	12.5	9.1
8	217.6	–	321.3	79.3	19.5	13.4
9	202.6	187.9	135.2	55.3	15.4	8.7
10	199.3	–	91.0	73.3	19.0	10.2
11	218.8	173.6	108.7	85	15.6	9.9
12	185.4	210.2	76.3	43.5	19.4	11.2
13	224.1	–	94.8	65.2	16.3	11
14	185.6	–	87.1	58.5	11.4	7.6
15	201.4	–	48.3	58.5	19.5	12.5
16	198.1	–	93.0	41.1	15.4	6.6
17	204.7	–	94.6	63.7	16.0	9.3
18	186.3	–	125.5	71.0	14.4	7.7
19	238.8	–	172.2	97.2	13.1	7.2
Mean	205.6^*^		129.9	68.1	16.0	10.2

–*, Not applicable.*

* *Mean of 24 hemisphere modeling (14 unilateral hemisphere with 5 bilateral hemispheres)*.

### Model Establishment Time

**Figure 2:**
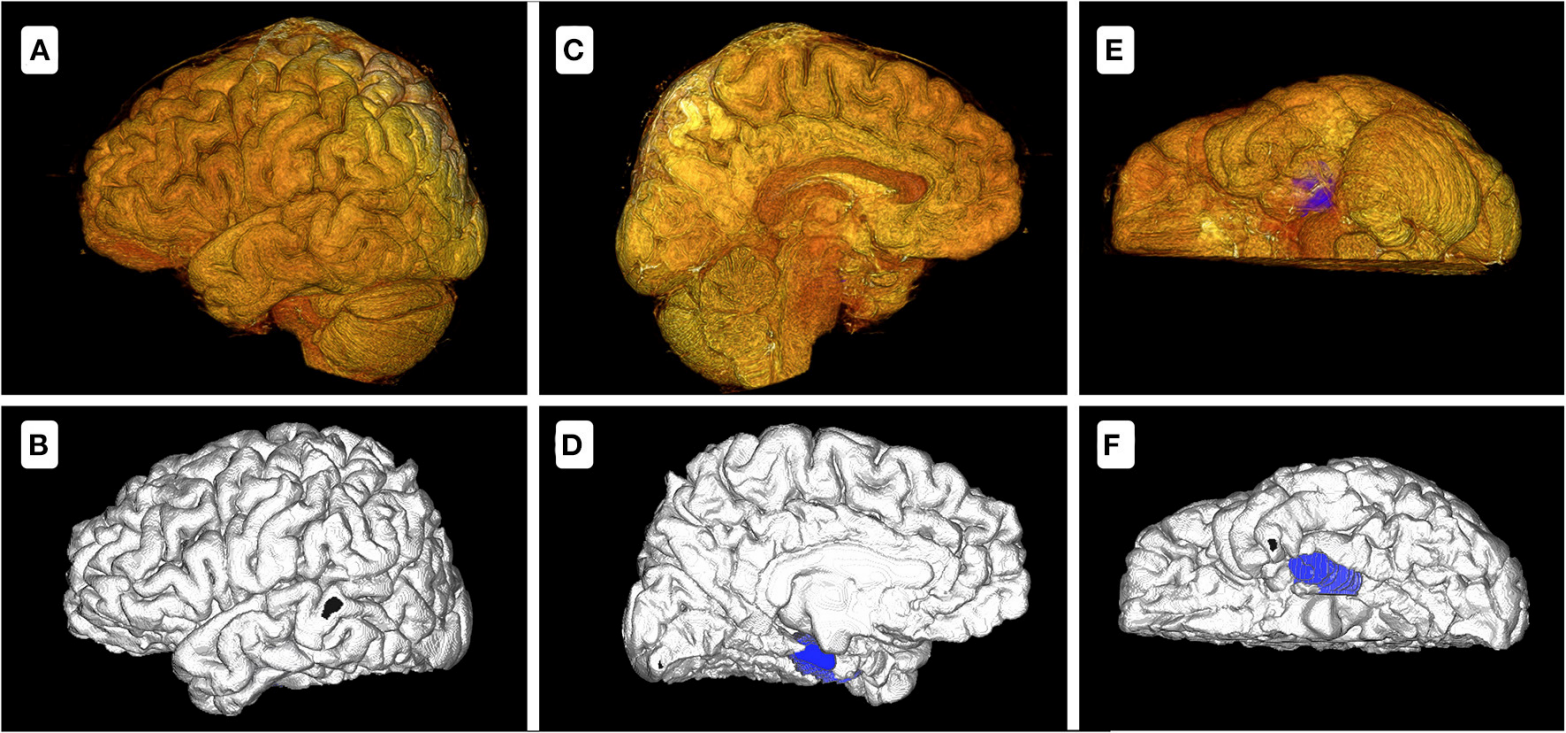
Model comparison between methods 1 and 2 of patient 6 with a lesion on the left medial temporal lobe. **(A,C,E)** show the cerebral-hemisphere and lesion models designed with method 1. **(B,D,F)** show the cerebral-hemisphere and lesion models designed with method 2. There are apparent differences between the two methods. In method 1, the sulci and gyri of the medial and inferior sides are unclear, with the blocking of cerebellum and brainstem, while method 2 does not exhibit these problems. In **(D,F)**, the blue demarcations represent the lesion model. In **(C)**, the lesion model is entirely blocked by the cerebellar hemisphere and brainstem. In **(E)**, the lesion model is partially blocked.

As outlined above, we designed the cerebral-hemisphere models using two methods. Twenty-four hemispheric models were established, including14 and 5 cases of unilateral and bilateral electrode implantation, respectively. A cerebral hemisphere model was designed in 175.3–238.8 s (mean, 205.6 s) and 20 s with methods 1 and 2, respectively (*p* < 0.05). There were some differences in the accuracy of the cerebral-hemisphere models established using these two methods, especially in terms of the medial and ventral cerebral structures, with the cerebellar hemisphere and brain stem blocking the field of vision. Method 2 accurately delineated these structures (Figure 2). The models identified a mean of 1.4 lesions (range, 1–5) in each patient. Significantly more time was needed to construct the model with method 1 (mean, 129.9 s; range, 48.3–435.9 s) than with method 2 (mean, 68.1 s; range, 50.1–103.8 s; *p* < 0.05).

### Electrode Placement Plan Time

**Figure 3:**
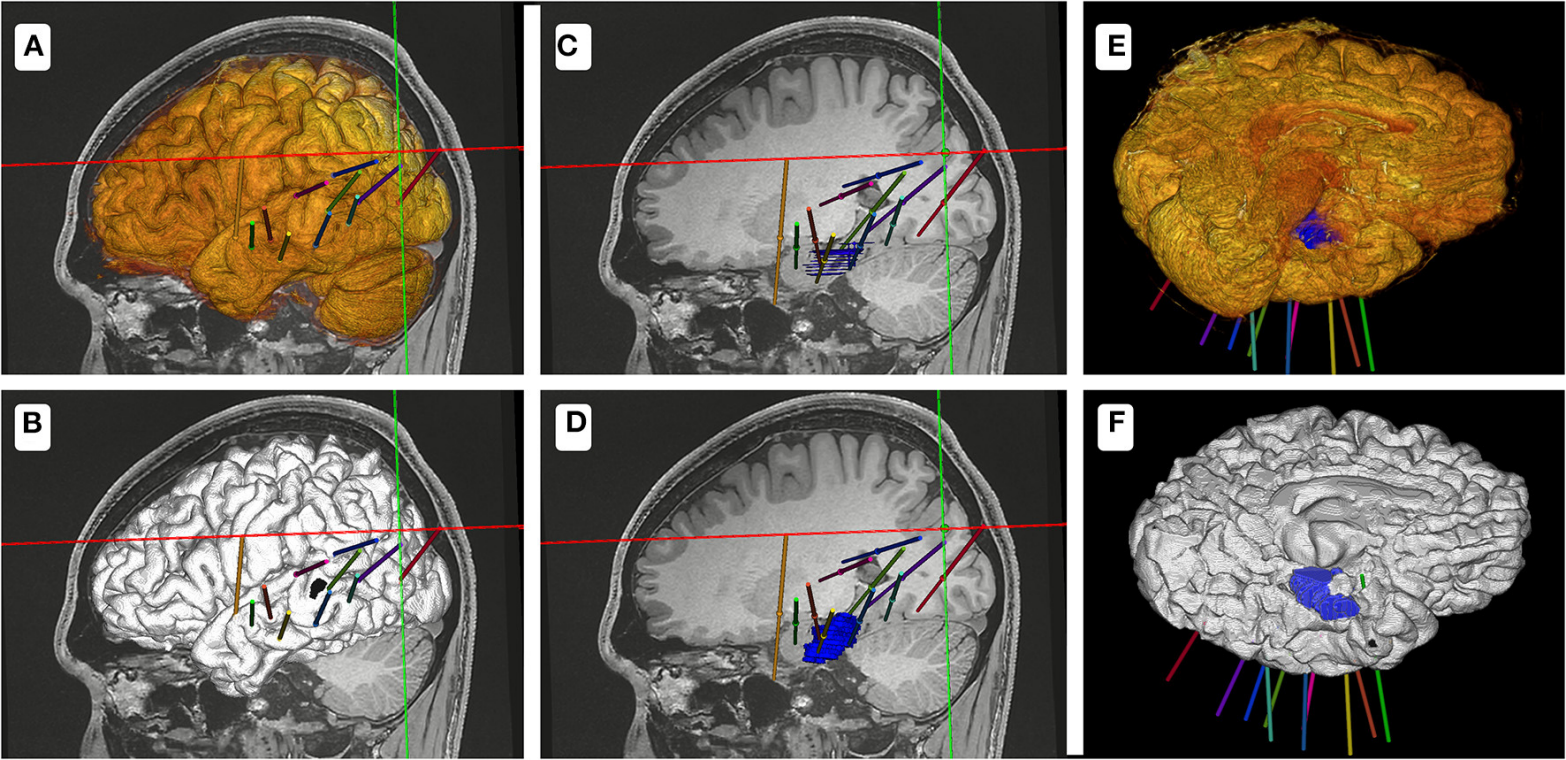
Schematic diagram of the two methods for the electrode-implantation plan. **(A,C,E)** show the electrode-implantation plan based on the model designed using method 1. **(B,D,F)** show the electrode-implantation plan based on the model designed using method 2. Because of the high resolution of the model designed using method 2, the design process of the electrode plan is relatively fast.

A total of 9–16 (mean, 11.8) electrodes were implanted in each patient. Methods 1 and 2 yielded electrode-implantation plan times of 9.1–25.5 min (mean, 16.0 min) and 6.6–14.8 min (mean, 10.2 min), respectively (*p* < 0.05). In 15 patients who underwent implantation for single lesions, more time was needed to construct an electrode-implantation plan using method 1 (mean, 15.3 min; range, 9.1–21.8 min) than using method 2 (mean, 9.8 min; range, 6.6–13.9 min; *p* < 0.05). Method 1 showed insufficient model definition and obscured lesions (Figure 3).

### Implantation Accuracy and Safety

Electrode-implantation surgeries were conducted based on the implantation plan designed using method 2. The average TPE of 231 electrodes amounted to 1.90 mm ± 0.37 mm (range, 0.33–3.61 mm). The average EPE was 0.89 ± 0.26 mm (range, 0.17–1.67 mm). None of the patients presented with intracranial hemorrhage or infection, and no other serious complications were observed.

## Discussion

Neurosurgical robots are currently commonly used in clinical practice, including the robotic stereotactic assistance (ROSA) and the Sinovation robotic system (Sinovation) (Zhao et al., 2020; Bonda et al., 2021). They are widely used in SEEG, deep brain stimulation, and other functional neurosurgical operations. The consistency and accuracy of the hardware have been demonstrated (Abel et al., 2018; Lu et al., 2021). However, the planning software of these systems has some limitations, particularly in terms of cerebral-hemisphere and lesion modeling. Current surgical planning systems do not include adequate and powerful modeling functions to meet the requirements of identifying individual differences in brain structure. To some extent, the lack of powerful modeling capabilities hinders the design of adequate SEEG electrode-implantation plans and may increase the likelihood of missed lesions. In this study, we found that the resolution of the brain model had an impact on the efficiency of the surgical planning. High resolution brain and lesion model could be reconstructed with combined use of FreeSurfer and 3D Slicer than with the routine robotic planning software, which in turn substantially reduced the time devoted to construct an adequate electrode-implantation plan. To our knowledge, this study was the first to combine these two approaches and proved their feasibility and efficacy in clinical work, and we found that the resolution of the brain and lesion model comprehensively affected the quality of the robot-assisted SEEG surgery.

### Speed of Model Reconstruction

While FreeSurfer requires relatively long operating times, the program runs and segments data automatically with a single line of code. The time required to initiate the program was ~10 s. 3D Slicer is equipped with more than 20 modeling tools, including advanced editing tools, such as islands, logical operators, and smoothing. In contrast, the modeling tools in robotic surgical planning systems only include simple functions, such as thresholding and segmentation. As such, it is much more difficult to design models with robotic surgical planning systems than with auxiliary software because SEEG surgery also pays attention to the brain region positioned beyond the isolated trajectory planning. Our analysis of modeling times demonstrated that FreeSurfer and 3D Slicer required much less time than the robotic surgical planning system to design a highly customized electrode-implantation plan.

### Model Resolution

As we know, there were several open-source automatic toolkits for brain model reconstruction, such as Brain Extraction Tool in FSL and Swiss Skull Sripper in 3D Slicer (Smith, 2002). The processing time of these toolkits were short, but the resolution of models was low, and there was no information about the medial side of the cerebral hemisphere. The two methods differed in the time needed to construct a brain model and the resolution of the model.

FreeSurfer accurately modeled both side of cerebral hemisphere, including the sulcus–gyrus structures in the medial and inferior side, for example, cingulate gyrus and lingual gyrus. The brain model reconstructed in method 1 had a high resolution on the lateral surface, but the medial and inferior surfaces were unclear (Figure 2). It was difficult to remove the cerebellum by the clipping tool because the cerebellum was too close to the occipital lobe. The lesion models designed using the assistive software were also more clearly because of the inherent limitations of modeling tools in the surgical planning system. These 3D-view structures could facilitate electrode trajectory planning and helped avoid high-risk structures of brain. Without the lesion model, the electrodes would probably miss or fail to cover the lesion to the maximum extent, and even lead to the failure of plan.

Because the robotic system did not support importing the model directly, we had to convert the model into DICOM format before importing. However, the data conversion led to reduction in the clarity of the model. Correspondingly, two possible ways to preserve the vector features and clarity of the model are to adjust the robotic system to support the direct import of the model and to modify the robotic system to enable processing of the original high-resolution model.

### Surgical Planning Time

SEEG provides electrical, clinical, and anatomical information on patients with epilepsy. High resolution models help identify the entry and target points and the orientation of the electrode during implantation. Concerning surgical planning time, we found that method 2 required significantly shorter planning time than method 1, which is especially important for patients with multiple lesions because multiple lesions may substantially increase the planning time required for method 1. Planning times may be shortened by: (1) clear modeling of the sulcus–gyrus structures on the medial and inferior surfaces of the hemisphere, allowing for quick target point selection, and (2) accurate lesion modeling, which facilitates the identification of the electrode trajectory through the lesion.

### Limitations and Future Work

Despite its notable strengths, several shortcomings of method 2 should also be considered. FreeSurfer and 3D Slicer are open-source programs available for scientific research purposes, and we should remain vigilant of their routine clinical applications. Therefore, it is worth pursuing the acquisition of Food and Drug Administration approval of such medical research functions with high clinical benefits. Accordingly, one possible step toward clinical application and formal approval is to upgrade the robotic surgical planning software to become compatible with third-party auxiliary software to improve the safety and ease of surgery. Another possibility is to enhance the modeling functions of the robotic surgical planning software in such a manner that the whole robotic system at the level of both the hardware and software becomes a comprehensive treatment system compatible with the principles of precision medicine. For now, this method seems superior to current conventional robotic surgical procedures, exhibiting higher clinical value and benefit for the preoperative planning stage in general.

## Conclusions

Robot-assisted SEEG electrode implantation provides a hardware advantage rendering electrode implantation safer and faster compared to previous approaches with the stereotactic frame (Machetanz et al., 2021). Our approach provided a software advantage that further improved surgical planning time and the efficiency of implantation planning at the software level.

Based on the results of this small sample-size study, FreeSurfer and 3D Slicer-assisted SEEG implantation seemed to provide valuable tools to enhance modeling speed and resolution, shorten the electrode-implantation planning time, and boost the efficiency of clinical work.

## Data Availability Statement

The original contributions presented in the study are included in the article/supplementary material, further inquiries can be directed to the corresponding author/s.

## Author Contributions

QL, XY, and JX determined the surgical plans. QL performed the operations. CW and JX helped perform the operations. QL and CW performed the statistical analysis. QL and JW wrote the first draft of the manuscript. QL, JW, and FW wrote sections of the manuscript. All authors contributed to manuscript revision, read, and approved the submitted version.

## Funding

This work was supported by Shanghai Jiao Tong University Fund for Interdisciplinary Research for Medical Applications (YG2021QN30).

## Conflict of Interest

FW was employed by Wuhan Zhongke Industrial Research Institute of Medical Science Co., Ltd. The remaining authors declare that the research was conducted in the absence of any commercial or financial relationships that could be construed as a potential conflict of interest.

## Publisher's Note

All claims expressed in this article are solely those of the authors and do not necessarily represent those of their affiliated organizations, or those of the publisher, the editors and the reviewers. Any product that may be evaluated in this article, or claim that may be made by its manufacturer, is not guaranteed or endorsed by the publisher.
